# The cerebellum ages slowly according to the epigenetic clock

**DOI:** 10.18632/aging.100742

**Published:** 2015-05-11

**Authors:** Steve Horvath, Vei Mah, Ake T. Lu, Jennifer S. Woo, Oi-Wa Choi, Anna J. Jasinska, José A. Riancho, Spencer Tung, Natalie S. Coles, Jonathan Braun, Harry V. Vinters, L. Stephen Coles

**Affiliations:** ^1^ Human Genetics, David Geffen School of Medicine, University of California Los Angeles, Los Angeles, CA 90095, USA; ^2^ Biostatistics, School of Public Health, University of California Los Angeles, Los Angeles, CA 90095, USA; ^3^ Pathology and Laboratory Medicine, UCLA David Geffen School of Medicine, Los Angeles, CA 90095, USA; ^4^ Center for Neurobehavioral Genetics, University of California, Los Angeles, CA 90095, USA; ^5^ Department of Internal Medicine, H.U. Marqués de Valdecilla-IFIMAV-University of Cantabria, Santander 39008, Spain; ^6^ UCLA Molecular Biology Institute; Department of Chemistry and Biochemistry; Los Angeles, CA 90095, USA

**Keywords:** tissue aging, brain, epigenetics, biomarker of aging, centenarian

## Abstract

Studies that elucidate why some human tissues age faster than others may shed light on how we age, and ultimately suggest what interventions may be possible. Here we utilize a recent biomarker of aging (referred to as epigenetic clock) to assess the epigenetic ages of up to 30 anatomic sites from supercentenarians (subjects who reached an age of 110 or older) and younger subjects. Using three novel and three published human DNA methylation data sets, we demonstrate that the cerebellum ages more slowly than other parts of the human body. We used both transcriptional data and genetic data to elucidate molecular mechanisms which may explain this finding. The two largest superfamilies of helicases (SF1 and SF2) are significantly over-represented (p=9.2×10^−9^) among gene transcripts that are over-expressed in the cerebellum compared to other brain regions from the same subject. Furthermore, SNPs that are associated with epigenetic age acceleration in the cerebellum tend to be located near genes from helicase superfamilies SF1 and SF2 (enrichment p=5.8×10^−3^). Our genetic and transcriptional studies of epigenetic age acceleration support the hypothesis that the slow aging rate of the cerebellum is due to processes that involve RNA helicases.

## INTRODUCTION

Since it is difficult to study what one cannot measure, the development of suitable measures of biological age has been a major goal of gerontology [1, 2]. Many biomarkers of aging have been studied ranging from telomere length [3, 4] to whole-body function such as gait speed. DNA methylation (DNAm) levels are particularly promising biomarkers of aging since chronological age (i.e. the calendar years that have passed since birth) has a profound effect on DNA methylation levels in most human tissues and cell types [5-14]. Several recent studies propose to measure accelerated aging effects using DNA methylation levels [15-20]. While previous epigenetic age predictors apply to a single tissue, our recently developed “epigenetic clock” (based on 353 dinucleotide markers known as Cytosine phosphate Guanines or CpGs) applies to most human cell types, tissues, and organs [19]. Predicted age, referred to as DNA methylation age, correlates with chronological age in sorted cell types (CD4 T cells, monocytes, B cells, glial cells, neurons), tissues and organs including whole blood, brain, breast, kidney, liver, lung, saliva [19], and even prenatal brain samples [21]. The epigenetic clock is an attractive biomarker of aging for the following reasons: a) it is more highly correlated with chronological age than previous biomarkers [22, 23], b) it applies to most tissues, cell types, and fluids that contain human DNA (with the exception of sperm), c) it relates, to some extent, to biological age since DNAm age of blood is predictive of all-cause mortality even after adjusting for chronological age and a variety of known risk factors [24]. Similarly, markers of physical and mental fitness are also found to be associated with the epigenetic age of blood (lower abilities associated with age acceleration) [25]. Perhaps the most exciting feature of the epigenetic clock is the prospect of using it for comparing the ages of different tissues and cell types from the same individual [19]. While the mathematical algorithm lends itself for contrasting the ages of different tissues, it remains an open research question whether the results are biologically meaningful. To provide empirical data for addressing this question, we proceed with all due caution in this study.

## RESULTS

**Figure 1:**
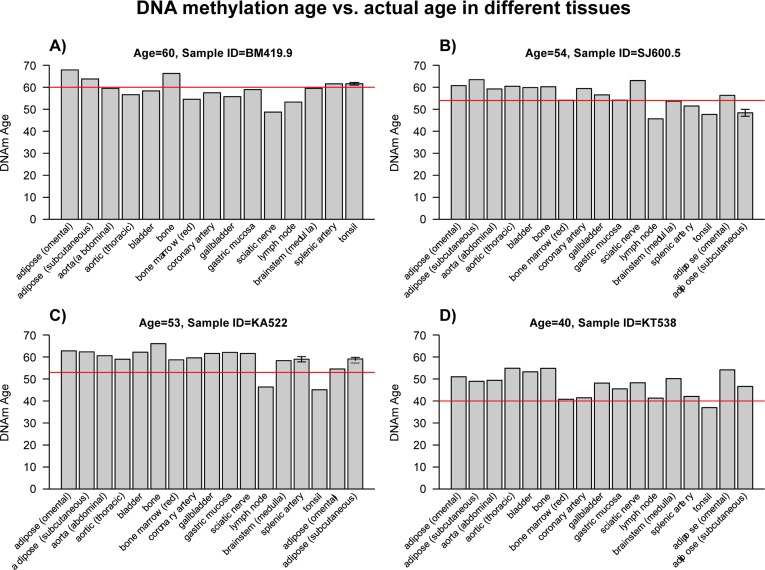
DNA methylation ages of various tissues from four middle-aged individuals Here we use data set 4 (Lokk, et al; 2014) to assess the tissue ages of 4 subjects (each of which corresponds to a different panel and person identifier such as BM419.9). Bars report the DNAm age in the corresponding tissue. The red horizontal line reports the chronological age. These plots confirm that tissues from the same middle aged individuals exhibit similar DNAm ages.

**Figure 2:**
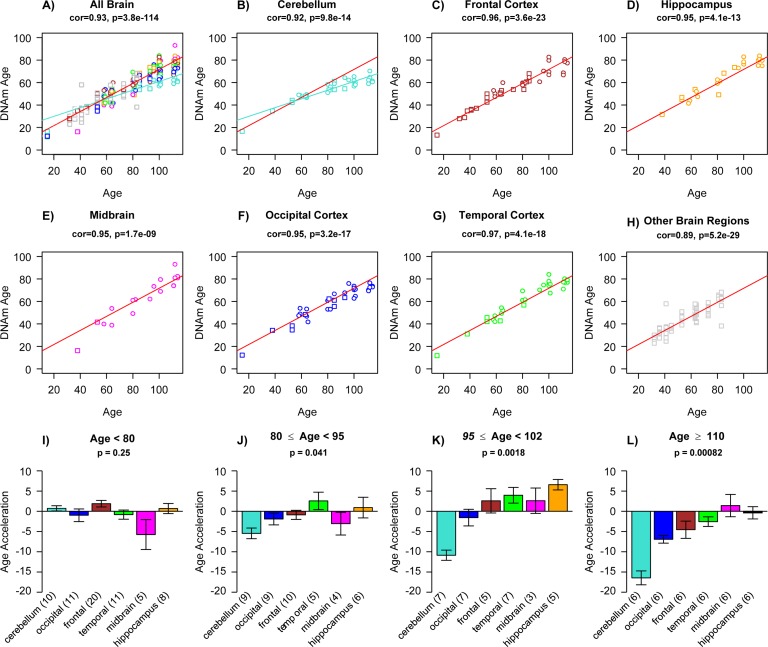
Epigenetic age acceleration in various brain regions (**a**) Scatter plot relating the DNAm age of each brain sample (y-axis) versus the corresponding chronological age (x-axis). Points are colored by brain regions (e.g. turquoise for cerebellum) as indicated in (**b-h**). Linear regression lines through cerebellar samples and non-cerebellar samples are colored in turquoise and red, respectively. Note that cerebellar samples (turquoise points) exhibit a lower rate of change (i.e. slope of the turquoise line) than non-cerebellar samples. In the scatter plots, circles and squares correspond to brain regions from Alzheimer's disease subjects and controls, respectively. Scatter plots show (**b**) cerebellar samples only, (**c**) frontal lobe, (**d**) hippocampus, (**e**) midbrain, (**f**) occipital cortex, (**g**) temporal cortex, and (**h**) remaining brain regions, which include caudate nucleus, cingulate gyrus, motor cortex, sensory cortex and parietal cortex. The subtitle of each scatter plot reports a Pearson correlation coefficient and corresponding p-value. Epigenetic age acceleration was defined as the vertical distance of each sample from the red regression line in (**a**). (**i-l**) Age acceleration versus brain region in different age groups as indicated in the respective titles. Cerebellar samples tend have the lowest (negative) age acceleration (turquoise bars) followed by occipital cortex (blue bars). Each bar plot depicts the mean value and one standard error and reports a non-parametric group comparison test p-value (Kruskal Wallis Test).

We had previously shown that tissues from the same middle aged individuals exhibit similar DNAm ages [19] and additional data from the public domain confirm this result (Figure 1). But it is not yet known whether some tissues collected from centenarians — and particularly supercentenarians appear to be younger than the rest of the body, which would indicate that they are better protected against aging. Here we assess the epigenetic ages of an unprecedented number of tissues (up to 30 tissues) from supercentenarians and younger controls. An overview of our data sets is presented in Table 1. Apart from three novel DNA methylation data sets, we also analyzed three publicly available data sets. Epigenetic age (referred to as DNAm age) was calculated as described in [19] from human samples profiled with the Illumina Infinium 450K platform. As expected, DNAm age has a strong linear relationship with chronological age in brain tissue samples (Figure 2a-h). We did not find a relationship between Alzheimer's disease (AD) and age acceleration in these samples from older subjects, which is why we ignored disease status in the analysis. Strikingly, the DNAm age of cerebellar samples exhibits a lower rate of change with age than non-cerebellar samples (as can be seen by comparing the turquoise line with the red line in Figure 2a,b). To formally measure age acceleration effects, we defined age acceleration as the residual resulting from a linear model that regressed DNAm age against chronological age in non-cerebellar brain sample. Thus, a tissue sample that exhibits negative age acceleration appears to be younger than expected based simply on chronological age.

**Table 1:** Overview of the DNA methylation datasets The rows correspond to the datasets used in this article. Columns report the tissue source, DNA methylation platform, number of subjects, access information and citation and a reference to the use in this text

Tissue source	Platform	No. arrays	No. subjects	No. females	Mean Age (range)	GEO/ArrayExpress ID	Citation	Figure
1. Brain	Illumina 450K	260	39	19	73 (15, 114)	GSE64509	Current article	2
2. Multiple tissues	Illumina 450K	64	1	1	112	GSE64491	Current article	3,4
3. Bone	Illumina 450k	48	48	46	78 (49-104)	GSE64490	Current article	6
4. Multiple tissues	Illumina 450K	70	4	1	52 (40, 6)	GSE50192	Lokk 2014	1
5. Brain+ blood	Illumina 450K	531	122	72	85 (40,105)	GSE59685	Lunnon 2014	5
6. Brain	Illumina 450K	87	46	16	62 (25,96)	GSE61431	Pidsley 2014	5

All brain regions have similar DNAm ages in subjects younger than 80 (Figure 2i), but brain region becomes an increasingly significant determinant of age acceleration in older subjects (as can be seen from the Kruskal Wallis test p-values in Figure 2i-l).

**Figure 3:**
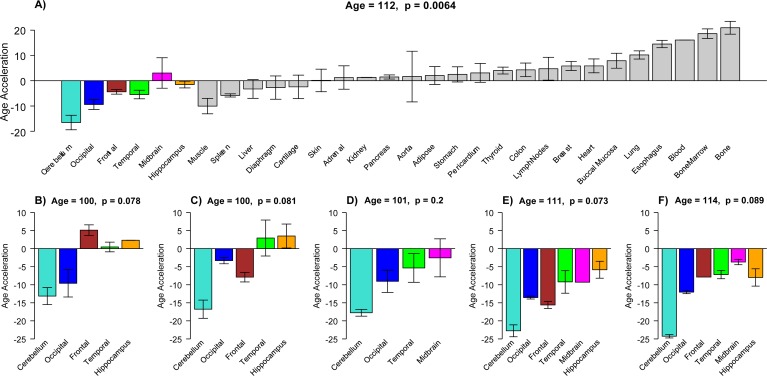
Epigenetic age acceleration in tissues from individual centenarians (**a**) Mean DNAm age acceleration per tissue (y-axis) for the 30 tissues and organs collected from a 112 year old woman. (**b-f**) Age acceleration in brain regions of 5 additional centenarians (whose age is in the title). Age acceleration here is defined relative to age of non-cerebellar brain samples as indicated by the red regression line in Figure 2a. Bars corresponding to different brain regions are colored as in Figure 2. For each of the six centenarians, cerebellar samples (turquoise bars) take on the lowest (negative values). Each bar plot reports the mean value and one standard error. Number of replicate measurements for each tissue was two except for bone and bone marrow, which were four.

Note that the cerebellum and to a lesser extent the occipital cortex exhibit negative epigenetic age acceleration in the oldest old (Figure 2k), i.e. these brain regions are younger than expected. These results can also be observed by focusing on six individual centenarians (Figure 3) and when evaluating two independent validation data sets (Figure 5).

### Comprehensive tissue analysis of a supercentenarian

**Figure 4:**
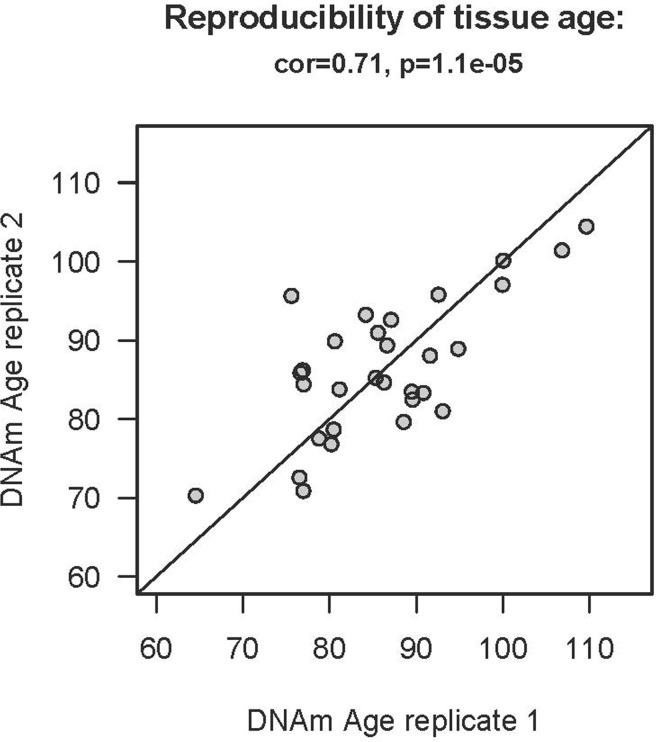
Reproducibility of DNAm age in the 112 year old supercentenarian For each of the 30 tissues of the supercente-narian, we assessed at least two replicates (two independent DNA extractions for distant regions of the same tissue).

To study age acceleration effects in non-brain tissues as well, we profiled a total of 30 tissues of a 112 year old woman (Figure 3a) who is described in Methods. We generated at least 2 replicate measurements per tissue and found that replicate age estimates are highly reproducible (r=0.71, Figure 4). Interestingly, the cerebellum exhibited the lowest (negative) age acceleration effect compared to the remaining 29 other regions. In contrast, bone, bone marrow, and blood exhibit relatively older DNAm ages. Given that bone appears to be older than other parts of the body, it is worth mentioning that our novel data demonstrate that the epigenetic clock applies to bone samples (largely comprised of osteocytes/osteoblasts) as well (Figure 6). To understand why the cerebellum evades epigenetic aging, we turned to transcriptional and genetic data.

**Figure 5:**
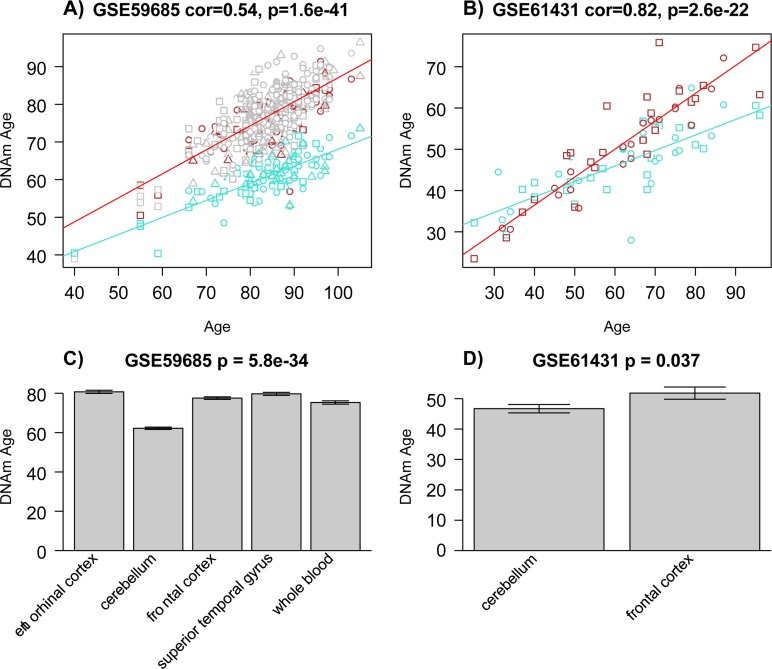
Epigenetic age acceleration in two multi-tissue data sets The first column **(a, c)** report results for samples from data set 5 [42]. The last column **(b, d)** reports findings for data set 6 [43]. **(a-b)** Scatter plots relating the DNAm age of each sample (y-axis) versus the corresponding chronological age (x-axis). Linear regression lines through cerebellar samples and non-cerebellar samples are colored in turquoise and red, respectively. Note that cerebellar samples (turquoise points) tend to lie below non-cerebellar samples. (**a**) Squares, circles, and triangles correspond to samples from controls, AD, and mixed dementia subjects, respectively. (**b**) Squares and circles corresponds to controls and schizophrenia subjects, respectively. (**c**) The barplots depict the mean DNAmAge (y-axis) versus tissue type for all subjects from panel A for whom all 5 tissue types (including whole blood) were available. (**d**) Analogous plot for all subjects from data set 6 for whom both brain regions were available.

**Figure 6:**
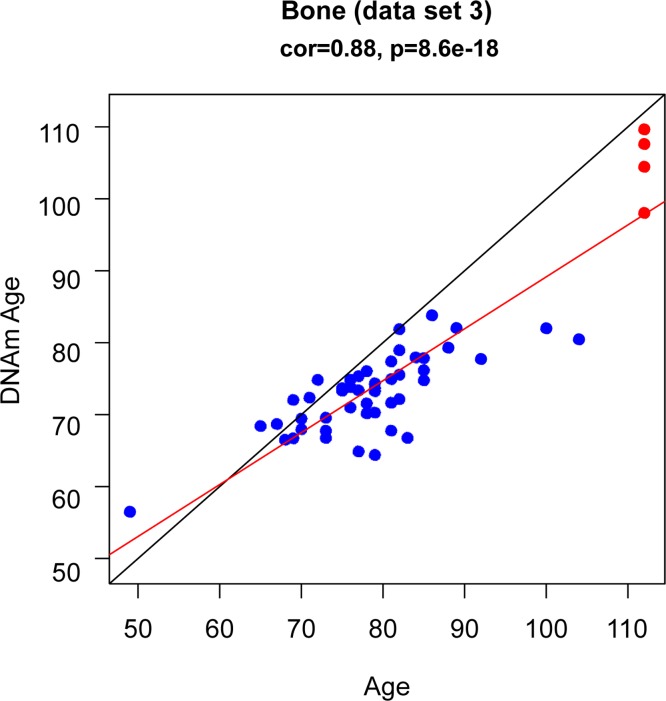
DNAm age (y-axis) versus age (x-axis) in bone (osteocytes/osteoblasts) The blue dots corresponds to the samples in data set 3 (bone). The red dots corresponds to the replicate bone samples from the 112 year old super centenarian.

### Characterizing gene transcripts that are over/under expressed in cerebellum

Using gene expression data from multiple brain regions of the Gibbs [26] data set (GSE15745), we identified 1239 gene transcripts that were significantly over expressed in cerebellum compared to the pons, temporal cortex and frontal cortex from the same subjects at a false discovery rate (FDR) of 0.05. The results of a functional enrichment analysis with the “Database for Annotation, Visualization and Integrated Discovery” (DAVID, v6.7) [27] can be found in Table 1. The 1239 over-expressed genes are highly enriched with genes that are located in the nucleus and are known to play a significant role in gene transcription, mRNA processing, RNA splicing and chromatin modifications.

Thirty one of these over-expressed genes are involved in helicase activity (Bonferroni corrected p-value=8.5×10^−6^). RNA and DNA helicases are considered to be enzymes that catalyze the separation of double-stranded nucleic acids in an energy-dependent manner often coupled to ATP hydrolysis. However RNA helicases can function in other roles such as RNA folding, ribosome biogenesis, anchoring of substrates to form ribonucleoprotein complexes as well as disruption of RNA-protein complexes [28]. Helicases have been classified into six superfamilies (SF1-SF6)[29, 30]. We find that the two largest superfamilies (SF1 and SF2) are over-represented among the 1239 gene transcripts (p=9.2×10^−9^) with enrichment of genes with helicase or ATP binding domains including DEAD/DEAH box domains (IPR014001). Specifically when we considered genes listed on rnahelicase.org that are involved in pre-mRNA splicing (AQR, SNRNP200, DHX8, DHX15, DHX16, DHX38, EIF4A3, DDX39B, DDX3X, DDX3Y, DDX5, DDX23, DDX42 and DDX46), seven of these 14 (AQR, SNRNP200, DHX16, DHX38, DDX5, DDX42 and DDX46) are significantly overexpressed in cerebellum compared to cerebral cortex (p = 1.28×10^−5^).

Similarly, we identified 808 gene transcripts that were under-expressed in cerebellum at an FDR threshold of 0.05. The top gene ontology categories among these under-expressed are specifically related to neuronal function and include neuron projection development, neuron differentiation, and synaptic transmission.

### Genetic enrichment analysis

To determine which of the cerebellum associated transcriptional differences might play a causal role in keeping the cerebellum young, we tested whether the gene categories from Table 2 show enrichment for SNPs that relate to epigenetic age acceleration in cerebellum. The MAGENTA approach [31] was used to test whether the sets of functionally related genes in Table 2 are enriched for SNP associations with epigenetic age acceleration in cerebellar samples. Toward this end, we applied MAGENTA to results from our GWAS meta-analysis of epigenetic acceleration in cerebellum (Methods). The meta analysis was based on four independent data sets for which both SNP data and cerebellar DNA methylation data were measured on the same subjects (n = 354, see Methods). The MAGENTA results can be found in the last column of Table 2. Even after adjusting for multiple comparisons, significant enrichment results can be observed for the GO category “nuclear lumen” (p=0.0016), and the molecular function “helicase activity” (p=0.0030). Helicase superfamilies SF1 and SF2 (particularly SF2) are highly enriched (p = 5.8×10^−3^) based on SNPs associated with the following genes DHX57, CHD8, DHX15, DDX19A, DDX19B, DDX2, BLM, SMARCA5, SNORA67, EIF4A1, HLTF, C9orf102. Interestingly, another DEAD box related gene, DHX16, was the most significantly (q-value=1.5×10^−5^) over-expressed gene in cerebellum compared to other brain regions but it was not implicated in our GWAS analysis.

**Table 2:** Functional enrichment of differentially expressed genes in the cerebellum compared to pons, frontal cortex and temporal cortex

Category	Term	DAVID			GWAS MAGENTA CRBM
1239 transcripts over-expressed in cerebellum		n	FE	P Bonf.	P-value
Cellular Compartment	nuclear lumen (GO:0031981)	202	2.5	6.0×10^−35^	1.6×10^−3^
	nucleoplasm (GO:0005654)	125	2.5	2.4×10^−20^	0.018
	nucleolus (GO:0005730)	101	2.6	1.7×10^−16^	0.014
	spliceosome (GO:0005681)	32	4.4	2.2×10^−9^	0.29
Biol. Process	transcription (GO:0006350)	265	1.9	9.5×10^−26^	0.15
	mRNA processing (GO_0006397)	55	3.4	1.4×10^−12^	0.38
	chromatin modification (GO:0016568)	55	3.1	4.5×10^−10^	0.026
Molecular F.	RNA binding (GO:0003723)	93	1.9	7.1×10^−7^	0.040
	helicase activity (GO:0004386)	31	3.3	8.5×10^−6^	3.0×10^−3^
INTERPRO	DEAD-like helicase, N-terminal (IPR014001)	31	4.4	9.2×10^−9^	5.8×10^−3^
**808 under-expressed in cerebellum**					
Biol. Process	synaptic transmission (GO:0007268)	39	3.1	2.2×10^−6^	0.50
	neuron differentiation (GO:0030182)	49	2.7	2.4×10^−6^	0.030
	neuron projection development (GO:0010975)	19	6.5	9.6×10^−7^	0.54

## DISCUSSION

While our study of a supercentenarian suggests that the epigenetic age of cerebellar tissue is younger than other part of the body we need to highlight several caveats. Although the epigenetic clock lends itself for comparing the epigenetic ages of multiple tissues, it remains to be seen whether the difference between nervous and non-nervous tissue reflects differences in biological aging rates. We are on safer ground when it comes to comparing the ages of different brain regions. We are confident in the finding that the cerebellum has a lower epigenetic age than other brain regions in older subjects since this effect could be observed in three independent data sets and 6 individual centenarians. This finding raises several questions. The most pressing question is whether this implies that the cerebellum is biologically younger than other brain regions? While the epigenetic age of blood has been shown to relate to biological age [24, 25], the same cannot yet been said about brain tissue. As a matter of fact, our study provides the first admittedly indirect and circumstantial evidence that the epigenetic age of brain tissue relates to biological age because the cerebellum exhibits fewer neuro-pathological hallmarks of age related dementias compared to other brain regions. But prospective studies in model organisms will be needed to show that the epigenetic age of brain tissue predicts the future onset of age related diseases even after correcting for chronological age and known risk factors. Another open question is why does the cerebellum have a slower aging rate? In an attempt to address this question, we used both transcriptional and genetic data. We found several gene ontology categories that are enriched in genes that are over-expressed in the cerebellum (including helicases). But the results from our differential expression analysis must be interpreted with caution for two reasons. First, cellular heterogeneity may confound these results since the cerebellum involves distinct cell types. Second, this cross-sectional analysis does not lend itself for dissecting cause and effect relationships. To partially address these concerns, we used genetic data. It is striking that SNPs that relate to the epigenetic age acceleration of the cerebellum also tend to be located near RNA helicase genes as observed in our transcriptional data. These results suggest that RNA helicase genes might play a role in slowing down the epigenetic age of the cerebellum. Unfortunately, RNA helicase genes are not a “smoking gun” for any particular molecular process. RNA helicases are ubiquitous and essential proteins for most processes of RNA metabolism (e.g. ribosome biogenesis, pre-mRNA splicing, translation initiation) and also function as regulators of gene expression by non-coding RNAs, detection of specific RNA molecules, sensing of small compounds or transduction of metabolic signals [32]. Although we could not find any prior literature on the role of RNA helicases in tissue aging, the same cannot be said for DNA helicases: e.g. WRN, which is a member of the RecQ helicase family, is implicated in Werner's syndrome, which is a recessively inherited progeria.

The interpretation of our main finding (regarding the epigenetic age of the cerebellum) is also complicated by the fact that we still don't know what is being measured by epigenetic age. While many articles suggest that age-related changes in DNAm levels represent random noise others suggest that there might be a purposeful biological mechanism [33-35]. DNAm age might measure the cumulative work of an Epigenomic Maintenance System (EMS) [19]. Under the EMS hypothesis, our findings suggest that cerebellar DNA is epigenetically more stable and requires less “maintenance work”. But many other explanations could explain our findings including the following: a) the cerebellum has a lower metabolic rate than cortex [36-38], b) it has far fewer mitochondrial DNA (mtDNA) deletions than cortex especially in older subjects [39], and it accumulates less oxidative damage to both mtDNA and nuclear DNA than does cortex [40].

In conclusion, this is probably the first study to show that the cerebellum ages more slowly than other brain regions and possibly many other parts of the body. By understanding why the cerebellum is protected against aging, it might be possible to understand the cause of tissue aging, which remains a central mystery of biology.

## METHODS

### Description of datasets listed in Table 1

All data presented in this article have been made publicly available in public repositories. Gene Expression Omnibus accession numbers are presented in Table 1.

### Data set 1

Bisulphite converted DNA from these samples were hybridized to the Illumina Infinium 450K Human Methylation Beadchip. 260 arrays were generated from 39 subjects (19 females). Twenty-one subjects presented with Alzheimer's Disease (AD) whereas 18 subjects did not have any neurodegenerative disease. None of the subjects had brain malignancies. After adjusting for chronological age, we could not detect an age acceleration effect due to AD status, which is why we ignored AD status in the analysis. We profiled the following brain regions: caudate nucleus (n = 12 arrays), cingulate gyrus (n=12 arrays), cerebellum (32), hippocampus (25), inferior parietal cortex (11), left frontal lobe (9), left occipital cortex (12), left temporal cortex (18), midbrain (18), middle frontal gyrus (12), motor cortex (12), right frontal lobe (20), right occipital cortex (21), right temporal cortex (11), sensory cortex (12), superior parietal cortex (12), and visual cortex (11).

### Data set 2

Multiple tissues from a 112 year old, female supercentenarian. 64 arrays were generated from 30 tissues/regions (listed in Figure 3).

### Data set 3

Novel bone data set. The trabecular bone pieces were obtained from the central part of the femoral head of Spanish (Caucasian) patients with hip fractures (due to osteoporosis) or subjects with osteoarthritis. Since osteoarthritis status was not related to DNA methylation age, we ignored it in the analysis.

### Data set 4

Multiple tissues (listed in Figure 1) from GEO: GSE50192 [41].

### Data set 5

Various brain regions and whole blood from (GEO data GSE59685) [42].

Specifically, the following tissues were available: entorhinal cortex, cerebellum, frontal cortex, superior temporal gyrus, and whole blood.

### Data set 6

Pre-frontal cortex and cerebellum samples from schizophrenics and controls (GEO data GSE61431) [43].

Disease status could be ignored in data sets 5 and 6 because our tissue comparisons involved samples from the same subjects. Our results were qualitatively the same after using a multivariate regression model that accounted for disease status.

### DNA extraction

AllPrep DNA/RNA/miRNA Universal Kit (Qiagen, cat # 80224) was used for the DNA extractions for frozen tissue samples. Cubes 3×3×3mm with approximate mass of ~30 mg were cut from histological specimens collected during necropsies. Bone was dissected into bone and bone marrow (3×3×3mm each specimen) and separated into 2 different microcentrifuge tubes for DNA extractions. The procedure was conducted on dry ice without thawing the samples down to preserve RNA quality for prospective studies. 30mg of frozen tissue was lysed with 600uL guanidine-isothiocyanate–containing Buffer RLT Plus in a 2.0mL microcentrifuge tube, and homogenized by using TissueLyser II (Qiagen) with 5mm stainless steel beads. Tissue lysate was continued with the AllPrep protocol for simultaneous extraction of genomic DNA and total RNA using RNeasy Mini spin column technology. DNA yields were on average 16ug, with the highest yield from Spleen tissue (46 ug) and the lowest yield from Adipose tissue (2.2 ug).

We did not use bone specimens where we could macroscopically see both solid bone and bone marrow, so we did not use any additional washing steps to remove bone marrow.

### Preprocessing of Illumina Infinium 450K arrays

In brief, bisulfite conversion using the Zymo EZ DNA Methylation Kit (ZymoResearch, Orange, CA, USA) as well as subsequent hybridization of the HumanMethylation450k Bead Chip (Illumina, SanDiego, CA), and scanning (iScan, Illumina) were performed according to the manufacturers protocols by applying standard settings. DNA methylation levels (β values) were determined by calculating the ratio of intensities between methylated (signal A) and un-methylated (signal B) sites. Specifically, the β value was calculated from the intensity of the methylated (M corresponding to signal A) and un-methylated (U corresponding to signal B) sites, as the ratio of fluorescent signals β = Max(M,0)/[Max(M,0)+Max(U,0) +100]. Thus, β values range from 0 (completely un-methylated) to 1 (completely methylated) [44].

Many authors have described methods for dealing with the two types of probes found on the Illumina 450k array [45-47]. This is not a concern for the epigenetic clock since it mainly involves type II probes. But our software implements a data normalization step that repurposes the BMIQ normalization method from Teschendorff [46] so that it automatically references each sample to a gold standard based on type II probes (details can be found in Additional file 2 from [19]).

### DNA methylation age and epigenetic clock

Many articles describe sets of CpGs that correlate with age in multiple tissues [5, 7, 8, 14, 48-50]. Although these reports firmly establish the strong effect of age on epigenetic modifications, individual CpG sites are unsuitable for global contrasting of the epigenetic ages of different tissues derived from the same individual. Epigenetic age was calculated as reported previously. The epigenetic clock is defined as a prediction method of age based on the DNAm levels of 353 CpGs. Predicted age, referred to as DNAm age, correlates with chronological age in sorted cell types (CD4 T cells, monocytes, B cells, glial cells, neurons) and tissues and organs including whole blood, brain, breast, kidney, liver, lung, saliva [19]. Mathematical details and software tutorials for the epigenetic clock can be found in the Additional files of [19]. An online age calculator can be found at our webpage (https://dnamage.genetics.ucla.edu).

### Finding gene transcripts that were differentially expressed in cerebellum compared to three other brain regions

The data set from Gibbs et al [26] also contained gene expression data from the brain regions of the same subjects for whom DNA methylation data were available. We used these data to find genes that were over-expressed in cerebellum compared to the pons, temporal cortex, and frontal cortex.

Since multiple brain regions were available for each subject, we used a paired T test to find genes that were differentially expressed between a) cerebellum and pons, b) cerebellum and temporal cortex, and c) cerebellum and frontal cortex. The matched design (3 brain regions from the same subjects) allowed us to condition out chronological age, ethnicity, gender, and other subject level confounders. For each of the three matched pairwise comparisons, we obtained a T-statistic based on the differences in expression values. Next we combined the resulting three T statistics using a conservative meta analysis approach: the scaled Stouffer method implemented in the “rankPvalue” R function [51, 52]. The resulting meta analysis p-values were transformed to local false discovery rates (q-values) using the *qvalue* R package [53]. At a 1-sided false discovery rate (FDR) threshold (qValueHighScale) of 0.05 we found 1239 Illumina probes that were over-expressed in cerebellum. Details on these and all other probes on the Illumina array can be found in Supplementary Table S1 (MarginalAnalysisGibbsMeta). Similarly we identified 808 gene transcripts that were significantly under-expressed in cerebellum at a FDR threshold of 0.05.

The results of a functional enrichment analysis with the “Database for Annotation, Visualization and Integrated Discovery” (DAVID, v6.7) [27] applied to the 1239 overexpressed and the 808 underexpressed genes can be found in Supplementary Table S2 (DavidEASEQ05over.xlsx) and Supplementary table S3 (DavidEASEQ05 under.xlsx), respectively.

Also our functional enrichment analysis results using DAVID are qualitatively unchanged when other FDR thresholds (e.g. 0.01) are used.

### MAGENTA analysis for GWAS enrichment

MAGENTA is a computational tool that tests for enrichment of genetic associations in predefined biological processes or sets of functionally related genes, using genome-wide association results as input [31]. MAGENTA is designed to analyze datasets for which genotype data are not readily available, such as large genome-wide association study (GWAS) meta-analyses. As input of MAGENTA, we used the results of a genome-wide meta-analysis for epigenetic age acceleration in human cerebellum. In total, this analysis involved cerebellar DNA methylation data and SNP data from 354 Caucasian subjects from the following independent studies: 59 Caucasian individuals from a study for Alzheimer's disease [42], 112 neurologically normal samples from [26], 147 samples from a case control of psychiatric disorders [54], and 36 Caucasian samples from a case control study of schizophrenia [43]. We ignored disease status in our GWAS analysis since it had a negligible effect on age acceleration in cerebellum (t-test *P* > 0.1). Caucasian ethnicity was verified in PLINK or EIGENSTRAT[55].

Age acceleration outcome measure was defined in the same way that we utilized the residuals from regression of DNAm age on chronological age. Quantitative trait association analysis was performed on each study, adjusted for principal components when necessary. Fixed-effects models weighted by inverse variance [56] were applied to combine the association results across studies, yielding a total of 4,586,301 association *P* values as the input for the MAGENTA analysis. We extended the gene boundary with +/− 50 kilobases to assign SNPs to their nearby genes and selected the GSEA (Genome Set Enrichment Analysis) method with cutoff set at 95^th^ percentile to estimate enrichment *P* values starting with 10,000 permutations then increased to 100,000 for *P* < 1.0×10^−4^.

### Brief Information of the 112 year old

The likely cause of death was bilateral organizing pneumonia. Neuropathologic findings were those of Alzheimer's disease, Braak stage IV-V, NIA-AA stage A2B2C2 [57]. Neuritic plaques were abundant in hippocampus, frontal cortex and temporal cortex and less prominant in basal ganglia and occipital cortex. Neurofibrillary tangles were abundant in hippocampus and sparse in frontal and temporal cortices.

### Ethics review and IRB

All subjects from the UCLA tissue bank signed the “Consent for Autopsy” form by the Department of Pathology at UCLA, and research procurement was performed under IRB Research Protocol Number 11-002504. Further, the epigenetic analysis is covered by IRB Research Protocol Number: 19119.

## SUPPLEMENTARY MATERIALS, TABLES










