# Supplementary material for: The cerebellum ages slowly according to the epigenetic clock
Source: Aging (Albany NY). 2015 May 11;7(5):294–306. doi: 10.18632/aging.100742 (PMC4468311; doi:10.18632/aging.100742)
Supplement: Supplementary file 1 [file aging-07-0294-s001.pdf]

SUPPLEMENTAL MATERIAL

Please browse the full text version to see the Supplemental Tables of this maniscript.

Supplemental Table S1. Meta-analysis of gene expression data from GSE15745 comparing cerebellum to frontal cortex, temporal cortex and pons using the WGCNA R package [52].

Supplemental Table S2. David Enrichment analysis results for all transcripts on the Illumina HT-12 array that are significantly over-expressed at a false discovery rate threshold of 0.05.

Supplemental Table S3. David Enrichment analysis results for all transcripts on the Illumina HT-12 array that are significantly under-expressed at a false discovery rate threshold of 0.05.

Supplemental Table S4. Detailed results for the GWAS enrichment analysis.
