# Supplementary material for: The cerebellum ages slowly according to the epigenetic clock
Source: Aging (Albany NY). 2015 May 11;7(5):294–306. doi: 10.18632/aging.100742 (PMC4468311; doi:10.18632/aging.100742)
Supplement: Supplementary file 5 [file aging-07-0294-s005.docx]

**Table S4 Detailed results for the MAGENTA analysis (GWAS enrichment)**

| **Database** | **Term** | **Description** | **No. genes*** | **Exp. no.**  **enriched genes** | **Obs. no.**  **enriched genes** | **Nominal *P*** |
| --- | --- | --- | --- | --- | --- | --- |
| GO | GO:0031981 | nuclear lumen | 1834 | 92 | 115 | 1.6x10^-3^ |
|  | GO:0006351 | transcription | 2626 | 131 | 137 | 1.5x10^-1^ |
|  | GO:0005654 | nucleoplasm | 1286 | 64 | 79 | 1.8x10^-2^ |
|  | GO:0005730 | nucleolus | 629 | 31 | 44 | 1.4x10^-2^ |
|  | GO:0016568 | chromatin modification | 438 | 22 | 31 | 2.6x10^-2^ |
|  | GO:0003723 | RNA binding | 1294 | 65 | 77 | 4.0x10^-2^ |
|  | GO:0004386 | helicase activity | 138 | 7 | 15 | 3.0x10^-3^ |
|  | GO:0010975 | neuron projection development | 238 | 12 | 12 | 5.4x10^-1^ |
|  | GO:0007268 | synaptic transmission | 638 | 32 | 32 | 5.0x10^-1^ |
|  | GO:0030182 | neuron differentiation | 1015 | 51 | 63 | 3.0x10^-2^ |
|  | GO:0006397 | mRNA processing | 388 | 19 | 21 | 3.8x10^-1^ |
|  | GO:0005681 | spliceosomal complex | 143 | 7 | 9 | 2.9x10^-1^ |
| INTERPRO | IPR001650 | DNA/RNA helicase, C-terminal | 102 | 5 | 12 | 4.3x10^-3^ |
|  | IPR014001 | DEAD-like helicase, N-terminal | 104 | 5 | 12 | 5.8x10^-3^ |
| RNA Helicase | Pre-mRNA  Splicing | 14 genes | 12 | 3 | 2 | 1.2x10^-1^ |
|  | Pre-mRNA  Splicing | 7 genes | 7 | 2 | 0 | 1.0x10^-1^ |

*the number of genes analyzed by MAGENTA.

Exp. no. enriched genes=expected number of genes with gene P values < 95^th^ percentile in entire genome.

Obs. no. enriched genes=observed number of genes with gene P values < 95^th^ percentile in entire genome.
